# Metal Sulphides and Their Carbon Supported Composites as Platinum-Free Counter Electrodes in Dye-Sensitized Solar Cells: A Review

**DOI:** 10.3390/ma12121980

**Published:** 2019-06-20

**Authors:** Edson Meyer, Asanda Bede, Nyengerai Zingwe, Raymond Taziwa

**Affiliations:** 1 Fort Hare Institute of Technology (FHIT), University of Fort Hare, Private Bag, X1314, Alice 5700, South Africa; emeyer@ufh.ac.za (E.M.); nzingwe@ufh.ac.za (N.Z.); 2 Chemistry Department, University of Fort Hare, Private Bag, X1314, Alice 5700, South Africa; 3 Applied Science Department, Walter Sisulu University, Old King Williams Town Road, East London 5200, South Africa; rtaziwa@wsu.ac.za

**Keywords:** dye-sensitized solar cell, counter electrode, power conversion efficiency, metal sulphides, platinum

## Abstract

Energy sufficiency is a critical requirement for the economic prosperity of modern countries. Efficient harnessing of solar energy using technologies such as the dye-sensitized solar cell could solve the energy problem which persistently plagues developing countries. Despite having a simple operational procedure and modest power conversion efficiency of 13.8%, the dye-sensitized solar cell consists of an expensive platinum counter electrode which makes commercial success futile. Thus, this review intends to establish the progress researchers have attained in the development of sulphide based counter electrodes as alternatives to platinum, thereby lowering cost of production. Metallic sulphides are good electrocatalysts and cheap, hence, they possess the necessary requirements for effective functional counter electrodes. Furthermore, ternary metallic sulphides are known to exhibit higher efficiencies stemming from the synergistic effect produced by the co-existence of two metal ions in a crystal structure, which is believed to induce greater catalytic capability. Incorporation of metallic sulphides with carbon materials, which are exceptional electrical conductors, could potentially produce more efficient counter electrodes. In that regard, this review seeks to establish the effect recently developed composite counter electrodes comprising metallic sulphides and carbon-based materials have induced on the functionality of the counter electrode (CE).

## 1. Introduction

Every single day, the sun radiates more energy than the whole world requires per year [1]. However, despite its huge potential, solar energy is amongst the least-used electricity sources. The minimal contribution by solar to the electricity generation sector can be attributed to the high cost of installation, electricity generation relative to existing electricity prices, as well as the low photo-to-electricity conversion efficiency most solar technologies exhibit [2]. A study in South Arica found significant reductions in production costs and rising demand for solar panels resulted in a decline of the cost of electricity generation for a photovoltaic system from 5 Rand per kilowatt/hour (kWh) in 2011 to 0.62 Rand per kWh in 2017 [3]. The steep decline in cost makes solar energy very attractive compared to the rising cost of electricity generated from coal, which was estimated to have risen from 1 to 1.84 Rand per kWh during the same period of time [3]. To further advance the use of solar energy for electricity generation, the cost of solar panel production should be reduced either through improving the manufacturing process, implementing newer and advanced technologies or changing the composition of the solar cells to constitute cheaper materials. The most common technology used for solar exploitation are silicon solar cells with a power conversion efficiency of 24% [4]. Silicon solar cells are afflicted by the high cost of silicon processing, the large amount of silicon required to meet the threshold for adequate electricity generation, as well as an average power conversion efficiency [2]. These significant problems make electricity generation from solar energy cost ineffective, therefore, development of technologies that can be used to exploit solar power at a cheaper cost is a priority. Recent progress in thin-film technologies has shown that efficiencies for electricity generation from solar energy can be vastly improved [5]. One such thin-film technology is the dye-sensitized solar cell (DSSC) which is cheaper and uses a simple mechanism for solar power generation [6]. Despite all the outlined advantages, the DSSC consists of two expensive platinum group metals, namely, ruthenium in the dye as well as platinum in the counter electrode (CE). In order to meet the goal of reducing the cost of solar power generation and attaining parity of prices with other sources of electricity generation, replacements for these two expensive metals must be sought. Replacements for the platinum counter electrode should be cheap and exceed, or at the very least maintain, the same efficiency of 13.8% as the best performing platinum counter electrode [7]. As such, this review outlines the progress attained up to date in the development of a suitable CE for the DSSC. Catalysts such as transition metal alloys [8,9], sulphides [10] and carbon-based materials [11,12] have already been tried and tested as potential replacements for platinum in dye-sensitized solar cell counter electrodes yielding various results. Despite most non-metallic counter electrode materials being cheaper than platinum, their catalytic activity is significantly lower which results in poor photovoltaic parameters, although it would reduce the cost of production for the DSSC. Implementation of these materials would basically be trading relatively high efficiency for cost effectiveness. Since the dye is the most significant component of the DSSC through its photon to electrons generation process, thus, any significant improvement to the power conversion efficiency (PCE), short circuit current and open circuit voltage can only be attained through improving the function of the dye. Kakiage et al. [7] reported on the implementation of collaborative dye molecules in a bid to increase the photon conversion efficiency. In his work, Kakiage et al. [7] utilized a silyl and carboxy anchor dyes termed ADEKA 1 and LEG 4 respectively. Resultantly Kakiage produced the best performing dye-sensitized solar cell up to date with 14.7% PCE. The best performing DSSC was composed of an TiO_2_ photoanode, ADEKA 1-LEG 4 dye Co(phen)_3_(PF_6_^−^)_2_ electrolyte and an Au/graphene nanoplatelets (GNP) counter electrode. In the same report, Kakiage et al. [7] noted that the performance of the DSSC composed of the Pt counter electrode was lower at 13.8% PCE. The work conducted by Kakiage et al. [7] involved the use of gold- and platinum-based counter electrodes which are expensive and would do less to promote the commercialization of DSSCs. Furthermore, available results show that appreciable efficiency was attained when the counter electrodes were metal based. Research into metallic-based counter electrodes has shown that sulphides produce better efficiency parameters as compared to nitrides [13], phosphates [14], and oxides [15]. Power conversion efficiencies (PCEs) for most of the materials mentioned above have been inadequate, resulting in the synthesis of composite materials in order to meet efficiency requirements. The metal sulphide/carbon material composite is the most exciting because of the qualities each individual material gives to the composite [16]. Metal sulphides particularly molybdenum disulphide is well known for its electrocatalytic ability, thereby finding wide use in the oil refinery industries as catalyst for sulphur removal in fossil fuels [17]. Furthermore, metal sulphides are easily accessible, thus making them ideal and cost-effective replacements for platinum in DSSC CE. On the other hand, carbon-based materials such as graphite are equally cheap while providing excellent electrical conductivity [18], whereas the more advanced carbon materials like carbon nanotubes are expensive [18]. As such, this review seeks to highlight how effective metal sulphide/carbon material composites have performed as replacements for platinum. In addition, discussions on how synthesis methods and composition affected the performance of the counter electrode are provided. Operational procedures of the DSSC as well as its composition are detailed in this review. 

## 2. Working Principle

The DSSC consists of four main components which are: the dye-sensitizer, mesoporous semiconducting photoanode, electrolyte, and counter electrode [6]. Under illumination from the sun, dye molecules (S) in the highest occupied molecular orbital (HOMO) absorb photons from the sun and their electrons become excited. These excited electrons (S*) subsequently move to the lowest unoccupied molecular orbital (LUMO) of the dye, provided the energy gained from the photons is large enough to overcome the energy difference between the HOMO and the LUMO (i). The excited electrons (e^−^) are then injected into the conduction band of the titanium dioxide photoanode (ii), diffuse through it and are subsequently led to the external circuit where they migrate towards the counter electrode (iv). At the counter electrode, the electrons are stripped (v) by the oxidized triiodide ion (I_3_^−^) in the iodine electrolyte, thereby itself being reduced to the iodide ion (I^−^). The reduced iodide ions then transfer the electrons back to the oxidized dye (vi) molecule reducing it in the process while becoming oxidized back to the triiodide ion. The transfer of electrons from the counter electrode to the dye by the iodide ion, thus, facilitates regeneration of the dye molecule and completion of the cycle of sunlight conversion into electricity. The difference in potential between the Fermi level of the nanocrystalline semiconductor photoanode and the redox potential of I^−^/I_3_^−^ gives the open circuit voltage. The operational cycle of the DSSC can be summarized by the following equations:

S + hv → S^*^; sunlight absorption (1)

S^*^→ S^+^ + e^−^; electron injection into the photoanode conduction band (2)

S^+^ + 3/2 I^−^ →S + 1/2 I_3_^−^; dye regeneration (3)

1/2 I_3_^−^ + e^−^ → 3/2 I^−^; iodine electrolyte regeneration (4)

**Figure 1:**
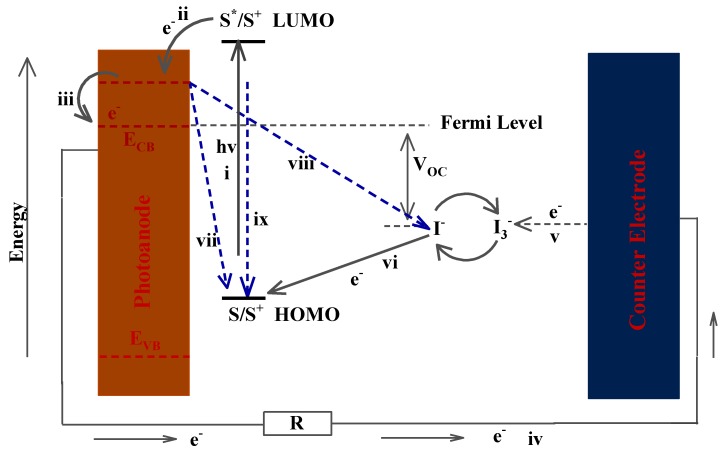
Illustration of the operational mechanism of the dye-sensitized solar cell. HOMO: highest occupied molecular orbital; LUMO: lowest unoccupied molecular orbital (hv—energy, E_CB_—conduction band energy, R—resistance, V_OC_—open circuit voltage. Reproduced with permission from Reference [19], MDPI 2018.

The operational cycle for a DSSC is illustrated in Figure 1.

## 3. Counter Electrode

**Figure 2:**
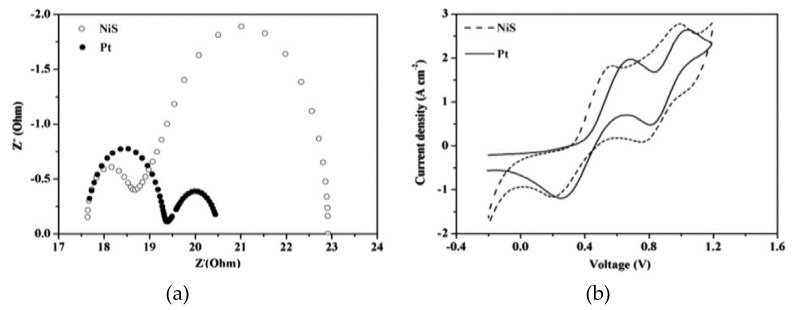
(**a**) Electrochemical Impedance Spectroscopy (EIS) and (**b**) Cyclic Voltammetry (CV) analysis for NiS and Pt counter electrodes. Reproduced with permission from Reference [22], Elsevier 2016.

The DSSC CE serves two critical functions which are to facilitate the reduction of the electrolyte, thereby ensuring the continued generation of electricity as well as being the conduit for electron transfer from the outer circuit back to the electrolyte [20]. In order for the CE to undertake its duties effectively, it requires a catalytic material that can simultaneously enhance efficient reduction of the electrolyte while being an excellent electron conductor [19]. Since platinum can offer both qualities, it was the preferred catalyst of choice in the first assembled DSSC. However, platinum being such a versatile catalyst is widely used in the motor, pharmaceutical and oil refinery industries, etc. As a result of the high demand, the price for platinum is astronomically high. For all the well-established industries mentioned above, the price of platinum is economical since significant revenues are generated, thereby mitigating the expensiveness of platinum compared to the newer dye-sensitized solar cell technology. Consequently, other alternatives have had to be sought which are cheaper and can offer the same performance as the platinum CE. Furthermore, the platinum counter electrode undergoes significant corrosion in the iodine electrolyte resulting in efficiency decline. Despite being catalytically active, platinum metal exhibits modest charge transportation capability which results in high charge transfer resistance. Poor electron transportation by all four components of the dye-sensitized solar cell leads to recombinations between the generated electrons and their holes in the dye, and the triiodide ion, thus, lowers the power conversion efficiency. Consequently, all the materials incorporated in the dye-sensitized solar cell should exhibit good-to-excellent electron conductivity. Cheap metallic sulphides with excellent catalytic activity and conductivity make for good DSSC CE. Metallic sulphides are attractive as possible replacements to the platinum counter electrode due to their cheapness as well as modest catalytic capability. Metallic sulphides are more catalytically active because of the synergistic effect created by the metal and sulphide atoms. Initial research into the influence of sulphides on counter electrodes was explored on binary sulphides yielding varied results. Han et al. [21] developed PbS, Ag_2_S, CuS, CdS, and ZnS counter electrodes which performed better at 6.49, 6.11, 5.29, 2.45, and 1.76% PCE as compared to 3.86% for the platinum counter electrode developed under similar conditions. Charge transfer resistance for the sulphide counter electrodes decreased in the order ZnS > CdS > Pt > CuS > Ag_2_S > PbS at 59.37 > 40.56 > 12.94 > 6.94 > 5.32 > 5.12 Ω, respectively. The obtained results showed greater efficiency by the three sulphide counter electrodes PbS, Ag_2_S, and CuS as compared to the platinum counter electrode developed under similar conditions. Power conversion efficiency values reflect on the overall functionality of all its four components, thus, more effective determination of how the counter electrode affects the dye-sensitized solar cell can be made from the electrochemical analysis. The sole effect of the CE can be determined through analysis of the rate of progression of the reduction reaction occurring at the counter electrode. This is achieved through undertaking cyclic voltammetry, electrochemical impedance spectroscopy (EIS) and tafel polarization analysis. In this work, determination of the catalytic effectiveness of each developed counter electrode was made through evaluation of the resistance to electron transfer obtainable from cyclic voltammetry (CV) and EIS analysis as well as the PCE. Cyclic voltammetry analysis provides two valuable figures which describe the electrocatalytic capability of the counter electrode. These are the reduction current density and the peak-to-peak potential difference ∆E_pp_. The higher the peak reduction current density, the greater the rate of reduction occurring at the counter electrode, whereas ∆E_pp_ signifies the amount of over potential in the counter electrode. Generally, the standard electrochemical rate constant of a redox reaction is inversely proportional to ∆E_pp_, as such, its value should be minimum [19]. Zhou [22] synthesized a NiS CE which produced a low 7.39% power conversion efficiency (PCE). Figure 2 shows the CV and EIS graphs of the as-synthesized counter electrodes.

The ∆E_pp_ values for the two CEs at 364 mV and 446 mV for NiS and platinum, respectively, showed that the rate of the reduction reaction was much faster when the metal sulphide CE was used. Electrochemical impedance spectroscopy analysis undertaken to confirm the CV results produced a larger charge transfer resistance R_ct_ of 1.72 Ω for the Pt CE compared to 0.81 Ω for NiS. This result indicates that lesser impedance-to-electron movement occurred when NiS was used as the CE. Despite producing efficiency parameters higher than the Pt CE, this metal sulphide counter electrode cannot compare to the 24% efficiency associated with silicon-based solar cells. Table 1 shows the photovoltaic performances of various binary sulphides as counter electrodes in DSSC.

Table 1 outlines the various techniques utilized in the fabrication and development of binary and ternary sulphide counter electrodes. The method of synthesis is vital in producing materials with conforming morphologies, that is, they should have nanometer-size diameters and should exhibit limited aggregation. Nevertheless, the cost factor contributes significantly to the decision of which methods can experimentally be utilized for counter electrode material synthesis. Table 1 clearly shows that hydrothermal synthesis is the most utilized technique for counter electrode synthesis. Hydrothermal synthesis is attractive because of the high-quality crystals it produces. [29] Although the method makes use of the expensive autoclave as well as the inability to observe the growth of the crystals in real time, its products are of high quality as evidenced by the high PCE and low charge transfer resistance. The other methods such as bath deposition and solvothermal, do not require any specialized material; however, the reduced control of reaction conditions leads to lower photovoltaic parameters. 

**Figure 3:**
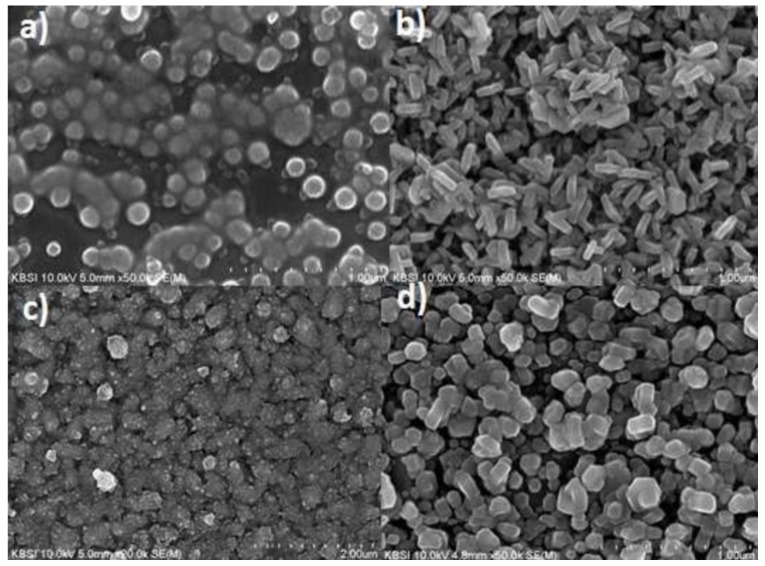
SEM images of NiS thin films deposited with different molar concentrations of thioacetamide. (**a**) Sample A, (**b**) Sample B, deposited with 0.4 M thioacetamide, (**c**) Sample C, (**d**) Sample D deposited with 0.8 M thioacetamide. Reproduced with permission from Reference [22] Elsevier 2015.

It is also evidently clear from Table 1 that most metal sulphides do perform well as CE in DSSC, as such, they possess the necessary qualities required for synthesis of effective CE. Efforts to improve efficiency by fabricating ternary and other multi-metallic sulphide counter electrodes have not yielded the desired product. Huo [10] synthesized a composite flower like nickel cobalt sulfide CE which was modified with NiS (NiCo_2_S_4_/NiS). The developed counter electrode only managed an 8.8% PCE with charge transfer resistance reaching 2.2 Ω∙cm^2^. Furthermore, a sputtering platinum like NiCo_2_S_4_ CE developed by Huang [30] exhibited an 8.1% PCE with 4.42 Ω∙cm^2^ charge transfer resistance. Although development of ternary sulphides increased the efficiency of the counter electrode as evidenced by the higher PCE and lower charge transfer resistance. The increases affected by the ternary sulphides were only marginal as compared to the 14.3% PCE for the platinum counter electrode. It should also be noted that the greater the multi-metallic sulphide to be fabricated, the more tedious and expensive the fabrication process becomes. Hence, it is evident that in the present form, sulphides only provide a cheaper option which does not meet the required efficiency to either replace the platinum counter electrode or to increase the commercial viability of the DSSC. As such, other alternatives should be sought. It is clear that higher power conversion efficiencies are required for the commercial success of the DSSC. Many factors affect the performance of a catalyst including the surface area and morphology of the catalyst. Surface area and morphology are two factors which, if efficiently manipulated at the nanoscale range, could lead to higher reduction rates since they ensure a higher rate of electrolyte adsorption as well as a wider area for adsorption [31]. In order to show how surface morphology and method of synthesis affects the performance of the CE, Punnoose et al. [32] undertook the synthesis of four NiS CE. Through varying the concentration of the reagents used in the fabrication of the CEs, Punnoose et al. [32] managed to produce varied morphologies ranging from nanoparticles to nanoplatelets. In order to evaluate how the variation in concentration affects surface morphology of the as-synthesized CEs, SEM analysis was conducted. Figure 3 shows the SEM images of the four synthesized NiS counter electrodes labelled Sample A–D. 

**Figure 4:**
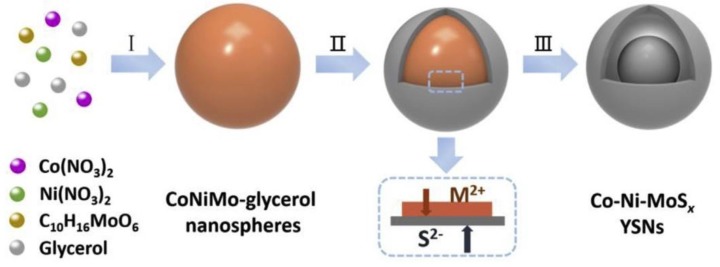
Schematic illustration of the procedure utilized for the preparation of CoNiMoS_x_. Reproduced with permission from Reference [36], Elsevier 2019.

Figure 3 clearly shows that variation in the concentration of the reagents used for fabrication led to varied surface morphologies. Figure 3a illustrates the agglomeration of nanoparticles in Sample A, whereas Sample B, consisting of nanowheat structures with a high surface area and uniform distribution, are shown in Figure 3b. The desirable properties of Sample B were attributed to the presence of triethanolamine. Upon increasing the concentration of thioacetamide from 0.4 to 0.8 M in the synthesized samples, a conversion of nanoparticles (Sample A) to nanoplatelets was observed. Sample D, fabricated without triethanolamine, showed a wider and uniform distribution of nanoplatelets compared to Sample C. Counter electrodes fabricated from well-distributed Samples A and D would be expected to perform better in DSSCs than those from Samples A and C, which are likely to be characterized by low charge transfer resistance, low ∆E_pp_, and higher PCEs. Samples A and B have the least charge transfer resistance R_ct_ with 3.64 and 5.22 Ω, respectively, compared to 11.62 and 7.15 Ω for Samples C and D. This result indicates greater conductivity by the nanoparticles compared to the nanoplatelets. The higher surface area and wider distribution of nanoparticles resulted in a higher rate of electrolyte adsorption, thus, greater catalytic activity was also experienced. This work also illustrates the problem associated with assigning CE performance solely on PCE values. Comparison of R_ct_ values for Samples A and D at 3.64 and 7.15 Ω, respectively, were indicative of greater electrical conductivity in Sample A, furthermore, open circuit voltage at 0.70 and 0.63 V indicated a higher electron movement when Sample A was used. However, the PCE values for the two samples showed that the DSSC using a counter electrode developed from Sample D performed better than Sample A. Therefore, parameters associated directly with the performance of the CE such as R_ct_ R_s_, and ∆E_ff_ are better indicators of measuring how a CE performs. From the work undertaken by Pannoose et al. [32], it is evidently clear that despite efforts to manipulate the morphology of the CE so as to attain higher catalytic performance, PCEs still remain low. Recent studies into the functionality of sulphide counter electrodes were conducted by Cho et al. [33], who developed the nickel–sulphide NiS_x_ counter electrode for DSSC applications. The developed sulphide counter electrode exhibited a 7.12% PCE compared to a platinum counter electrode fabricated under similar conditions. Ganesh et al. [34] also attempted the fabrication of carbon supported metals sulphide counter electrodes doped with nitrogen NiMoS-Nitrogen and CuMoS-Nitrogen. Despite all the enhancement, the efficiencies of the developed counter electrodes were 2.85% and 2.62% for NiMoS-NG and CuMoS-NG, respectively. These values were obtained despite the NiMoS-NG exhibiting a peak-to-peak potential difference of 382 mV, which is indicative of greater catalytic activity as compared to the platinum counter electrode with 450 mV. Hou et al. [35] fabricated an interconnected CoInS_4_ CE with a nanosheet structure, which produced PCE of 8.83% as compared to 8.19% for the platinum counter electrode. The CoInS_4_ counter electrode exhibited higher charge transportation with resistance being 2.39 Ω∙cm^2^ as well as decent catalytic activity indicated by its low peak-to-peak potential difference of 0.51 V to 0.56 V for platinum. The higher activity for the ternary sulphide counter electrode was ascribed to its interconnected nanosheet structure, which facilitated a greater area for catalytic activity and electron transportation. Further studies into multi-metallic sulphide-based counter electrodes were conducted by Qian et al. [36] through development of CoNiMoS_x_ yolk shell nanospheres for use as counter electrodes in DSSC. Figure 4 shows the procedure utilized by Quan et al. [36] to produce the yolk shell nanospheres. 

**Figure 5:**
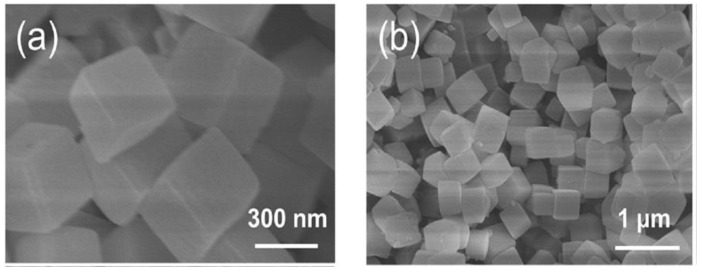
SEM images of the CoMoS_x_ nanocubes. (**a**) 300 nm magnification, (**b**) 1 µm magnification. Reproduced with permission from Reference [37], Elsevier 2018.

The developed ternary and quaternary yolk shell nanospheres exhibited high catalytic activity and electrical conductivity as indicated by the low charge transfer resistance and peak-to-peak potential difference for CoNiMoS_x_ at 0.37 Ω and 255 mV as compared to 2.38 Ω and 328 mV for platinum CE. The high catalytic activity resulted in an efficient photo to electricity conversion process which yielded a 9.12% PCE compared to 8.24% for the platinum counter electrode. The higher catalytic activity of the yolk shell nanospheres was credited to the coarser surface of the nanospheres, which increase surface area as well as the synergy created between the elements in the composite which potentially results in charge transfer from one element to another resulting in enhanced catalytic active sites on the outer surface. Additional studies in the functionality of sulphide-based counter electrodes were recently conducted by Xu et al. [37] through development of cobalt–molybdenum disulphide CoMoS_x_ nanocubes using a step template conversion process. The CoMoS_x_ counter electrode yielded an impressive 9.64% PCE compared to 8.39% for the platinum counter electrode. Figure 5 shows the morphology of the CoMoS_x_ nanocubes with an average size ≈ 300 nm. 

The catalytic activity of the nanocubes was significantly higher as indicated by the lower peak-to-peak potential difference of 280 mV as compared to 56 mV for platinum. Electron migration was resultantly higher for CoMoS_x_, with the charge transfer resistance being a lowly 1.70 Ω to 3 Ω for the platinum CE. As clearly shown, newer fabrication techniques have resulted in higher yield samples possessing morphologies that enhance the electrocatalytic capability of the sample as well as electron transfer process. These techniques could potentially be beneficial for the development of the counter electrode that exceeds the performance of the platinum counter electrode. Despite recent improvements and newer methods of metal-sulphide preparation the catalytic ability and PCE of the sulphide-based counter electrodes was still lower than for the best functioning platinum counter electrode at 13.8% PCE. Therefore, other alternatives to increase efficiency have to be sought. One such method is to fabricate composites with other materials which enhance the properties of the sulphide compound. Ideal materials that can be used for synthesis of sulphide composites are carbon-based materials, since they possess excellent electrical conductivity which is a necessity of charge transfer between the counter electrode and the electrolyte. Carbon materials have for a long time been known to be good electrical conductors and also possess excellent chemical stability [18]. They are especially attractive since they are abundant, easily accessible, and moderately cheap [18]. Also significant is the modest chemical activity of carbon-based materials which can be attributed to the high internal surface area of carbon atoms that offer, abundant catalytic active sites, thereby leading to a small charge transfer resistance [18]. A smaller charge transfer resistance signifies a higher rate of generation of iodide ions from the triiodide ions, resulting in higher PCEs. Table 2 illustrates some of the advantages associated with carbon-based materials.

Amongst all the carbon-based materials, graphene commands the most attention due to its specific properties which, if effectively harnessed and developed, could lead to significant improvement in the electronic and optical industries, cost and performance wise. Graphene consists of a single atomic layer composed of sp^2^-hybridized carbon. Due to its unique electronic configuration, graphene has high conductivity and transparency which is ideal for use as electrodes or interlayers in photovoltaic devices [12]. Excellent electrical conductivity by graphene stems from its high charge carrier mobilities averaging 200,000 cm^2^∙V^−1^∙s^−1^ compared to 980 cm^2^∙V^−1^∙s^−1^ for MoS_2_ [12]. The excellent conductivity by graphene is helpful for metallic sulphides which exhibit sufficient catalytic capability with relatively poor electrical conductivity as indicated by the high charge transfer values. In order for the synthesized counter electrode to effectively catalyze the reduction of the electrolyte, the maximum specific area in which adsorption of the electrolyte can take place should be available. With the specific surface area of a single layer of graphene equaling 2630 m^2^g^−1^, graphene certainly provides adequate surface area to enhance interaction between the electrolyte and the thin catalyst film attached on the counter electrode [18]. Theoretically, graphene possesses all the necessary properties to be a good counter electrode material; however, practical results in which graphene has solely been the counter electrode have produced mediocre performances. Sahito [38] fabricated a DSSC composed of graphene nanosheets as the counter electrode, while phenoxazine was used as the dye. The DSSC fabricated by Sahito [38] produced a low 6.61% PCE which was nevertheless higher than the 5.09% attained by the platinum counter electrode synthesized under similar conditions. Table 3 shows photovoltaic parameters for the most commonly used carbonaceous counter electrodes. Although they have exceptional electrical conductivity, most carbonaceous materials also exhibit poor catalytic activity as counter electrodes in DSSC characterized by low reduction current densities and PCEs. It is evident from Table 3 that the only feasible approach to developing efficient counter electrodes based on carbon materials is the incorporation of the various carbon materials into composites, since it leads to even lower charge transfer resistance and higher reduction current density. Charge transfer resistance for almost all the listed carbonaceous CE were very low, which suggests high electrical conductivity. This is an exceptional quality required for counter electrodes to facilitate unimpeded transfer of electrons from the outer circuit through the counter electrode to the electrolyte. The excellent electrical conductivity has led to most carbonaceous materials being used as supports in composites so as to shore up electron conductivity, as well as facilitating a large surface area for electrolyte contact with the counter electrode [39].

**Figure 6:**
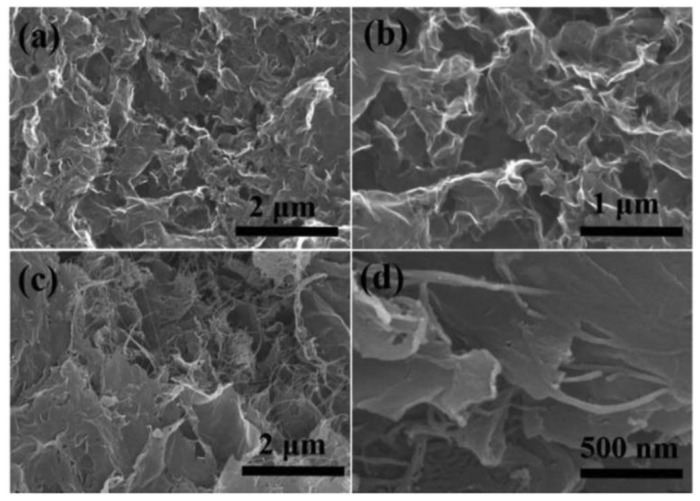
SEM micrographs for the RGO aerogels (**a**,**b**); GCT aerogels (**c**,**d**). Reproduced with permission from Reference [42], Elsevier 2019. (RGO—reduced graphene oxide, GCT—graphene/carbon nanotube composite).

Although most of the tabulated pure-carbon materials exhibited poor catalytic properties, their composites performed exceptionally well with the graphene/carbon nanotube (GCT) composite, producing a 20.76 mA∙cm^−2^ reduction current density. This graphene/carbon nanotube composite (GCT), which was synthesized by Yu [41], possessed a three-dimensional (3D) structure and exhibited a 10.69% PCE. This high PCE was attributed to the 3D structure of the CE which offered a large specific surface area, thereby ensuring higher rates of electrolyte adsorption and catalytic activity. Figure 6 shows a comparison of the SEM micrographs for the reduced graphene oxide (RGO) and the 3D graphene/CNT counter electrodes fabricated by Yu [41]. The SEM diagrams reveal thin graphene nanosheets which self-assemble into 3D loosely structured aerogels that randomly extended in various directions. 

According to Yu [41], the graphene/CNT (GCT) composite exhibited limited aggregation which was attributed to the cross linking, intertwining, and interspersion of single-walled carbon nanotubes in the graphene sheets. These activities are believed to support the graphene sheets and effectively expand the distance of the graphene sheets thereby weakening their curl and limiting them from being stacked by ᴨ–ᴨ bonds, thus, they exhibit less agglomeration. The electrochemical properties of the carbon composites show that less impedance to electron transfer was experienced when the GCT composite was used as compared to the platinum and reduced graphene oxide (RGO) CEs. Charge transfer resistance was minimal for GCT at 0.32 Ω compared to 0.57 and 0.74 Ω for the platinum and RGO counter electrodes, respectively. The lower charge transfer for GCT is indicative of its superior electrical conductivity, which facilitates faster dye regeneration thereby limiting charge recombination and ultimately improves power conversion efficiency. Cyclic voltammetry analysis for the three CEs clearly illustrated the excellent catalytic capability of GCT. Reduction current density (J_p_) was highest for RGO at 3.62 mA∙cm^−2^ compared to 3.61 and 3.57 mA∙cm^−2^ for GCT and platinum, respectively. This implies that both RGO and GCT are equally effective as electrocatalysts. Peak-to-peak potential difference (∆E_pp_), which signifies how fast the reduction reaction transpired, was highest for platinum at 327 mV compared to 224 and 236 mV for RGO and GCT, respectively. Furthermore, the charge transfer resistance (R_ct_) for RGO at 0.74 Ω was almost twice that for GCT, indicating that the addition of CNT to the reduced graphene oxide improves charge transfer between the counter electrode and electrolyte. According to Yu [41], the incorporation of single-walled carbon nanotubes which have a large specific surface area provides more catalytic sites for iodide reduction, thereby enhancing catalytic activity. Overall power conversion efficiency shows that the DSSC with the GCT counter electrode performed the best with 10.26% compared to 7.64 and 7.25% for platinum and RGO counter electrodes, respectively. The PCE values validate the electrochemical analysis data which outlined the superior catalytic activity exhibited by GCT compared to RGO and platinum counter electrodes. The work by Yu [41] clearly showed how carbonaceous materials could be viable platinum-free counter electrodes in DSSC. However, the high prices for all the best performing carbon materials listed in Table 4 show that, at the present moment, it would be uneconomical to fabricate counter electrodes from them. The prices of carbon nanotubes, reduced graphene oxide, and graphene are extremely higher than for platinum. In order for carbon materials to be considered as viable replacements for platinum, they have to significantly lower the cost factor which is the largest impediment to commercialization 

**Figure 7:**
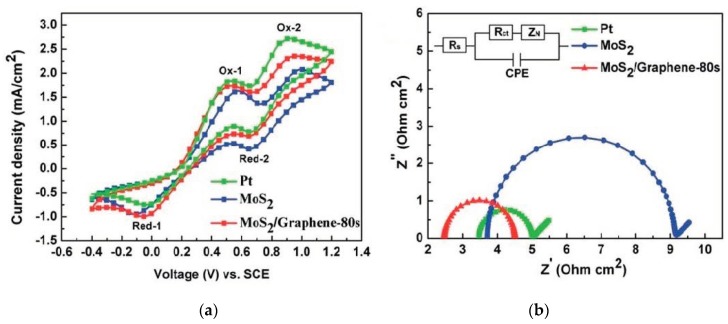
(**a**) Cyclic voltammetry and (**b**) EIS analysis for the MoS_2_/graphene composite. Reproduced with permission from Reference [49], RSC Publishing 2016.

The cheaper forms of carbon like graphite do not exhibit the desired electrochemical performance to be effective counter electrodes. According to Table 3, carbon black performs exceptionally well, while also being the cheapest carbonaceous material. All the same, the 9.1% efficiency exhibited by carbon black is still very low compared to the platinum efficiency as such incorporation of carbon materials into composites with other materials could potentially produce the required efficiency which would exceed the platinum and silicon cell efficiencies. Composites of carbon materials with metallic sulphides are especially attractive since sulphides are cheap as clearly illustrated in Table 5. Development of these composites could potentially mitigate the poor power conversion efficiencies of both metallic sulphides and carbonaceous materials through synergistic influence of each material in the composite. Ideally composites should consist of materials that complement each other. Since metallic sulphides are catalytically more active than graphene they will be tasked with enhancing the reduction reaction of the electrolyte whilethe carbon material offers greater electrical conductivity as well as a large specific surface area. To determine how effective metallic sulphide composites with carbon could be Li [49] fabricated a MoS_2_/graphene counter electrode which exhibited 8.01% PCE compared to 8.21% for the platinum counter electrode. In this composite MoS_2_ was tasked with enhancing the electrocatalytic capability of the composite while graphene ensured the availability of a large surface area for electrolyte adsorption as well as enhancing electrical conductivity. Cyclic voltammograms of the MoS_2_, MoS_2_/graphene, and Pt counter electrodes showed the existence of two pairs of redox peaks Ox-1/Red-1 and Ox-2/Red-2, indicating catalytic activity towards the reduction of the triiodide ion). Cyclic voltammetry (CV) analysis offers two valuable indicators of how effective the catalytic process proceeds. Figure 7 shows the cyclic voltammetry analysis results for the MoS_2_/graphene composite. The ∆E_pp_s for the three electrodes under investigation were 691 mV, 584 mV, and 572 mV corresponding to the platinum, MoS_2_/graphene, and MoS_2_ counter electrodes, respectively. This gives a clear indication of the increase in the rate of reaction when graphene is added to MoS_2_, albeit being lower than the platinum rate. Further evaluation of the cathodic peak reduction current densities at 1, 1.2, and 0.7 mA∙cm^−2^ for MoS_2_, MoS_2_/graphene, and platinum, respectively, validate the better efficiency of the composite compared to MoS_2_. To further elucidate on the effect of graphene on the composite, electrochemical impedance spectroscopy (EIS) measurements were undertaken. Charge transfer resistance R_ct_, which gives an indication of the electron movement occurring between the counter electrode and the electrolyte, shows that the composite MoS_2_/graphene performs better than the MoS_2_ counter electrode, with 3.71 Ω∙cm^−2^. Li [49] attributed the enhanced conductivity to the superior carrier mobility of graphene. Figure 7b shows the Nyquist plots obtained for the EIS measurements for the three counter electrodes. The R_ct_ semicircle for MoS_2_ was clearly larger than the other two counter electrodes indicative of its poor electrical conductivity. Charge transfer resistance for platinum was the least, as clearly shown by the smaller semicircle in the EIS diagram.

**Figure 8:**
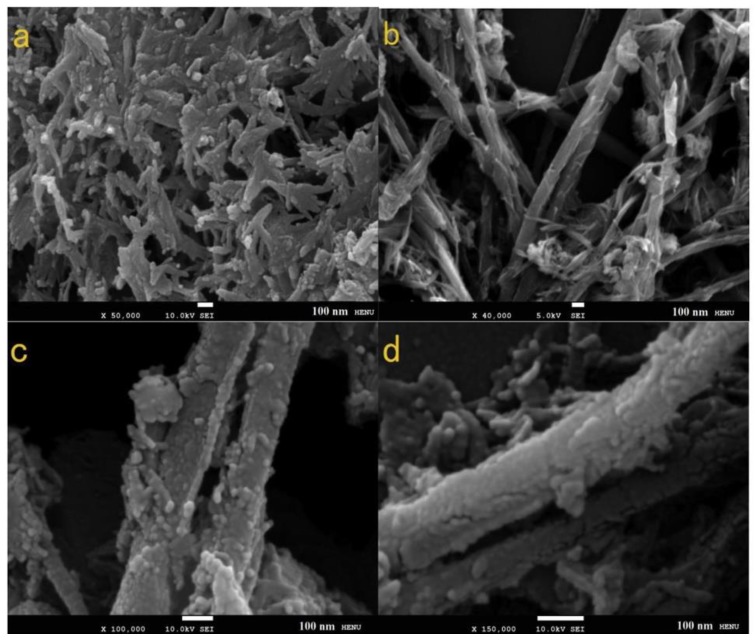
SEM micrographs for (**a**) VS_2_, (**b**–**d**) VS_2_/CNT. (**c**,**d**) Enlarged display of a VS_2_/CNT. Reproduced with permission from Reference [40], Elsevier 2017.

As illustrated in Table 2, carbon nanotubes do possess high electrical conductivity which is vital for electron transfer from the CE surface to the electrolyte, which directly impacts the catalytic capability of the CE. To ascertain how effective carbon nanotubes could be in composites with metal sulphides, Yue [39] undertook research to fabricate a hybrid vanadium sulphide CE decorated with carbon nanotubes. Synthesis of the VS_2_/CNT (vanadium sulphide/carbon nanotube) composite was conducted in a hydrothermal autoclave. Samples were generated at temperatures 140 °C, 160 °C, 180 °C, and 200 °C for 24 h, respectively. Morphologies of the synthesized VS_2_ and VS_2_/CNT are shown on the SEM diagrams in Figure 8.

**Figure 9:**
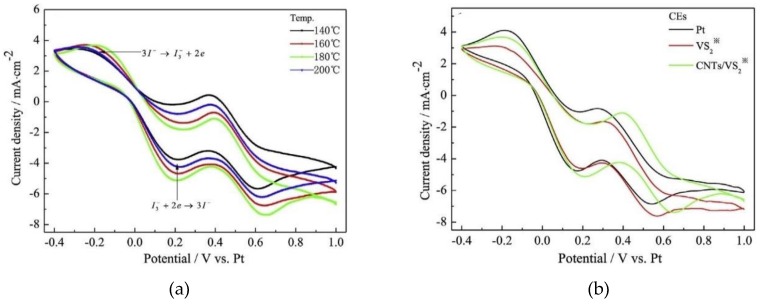
Cyclic voltammetry curves for (**a**) VS_2_/CNT counter electrodes (CEs) synthesized at different temperature conditions, (**b**) various CEs with a scan rate of 50 mVs^−1^. Reproduced with permission from Reference [39], Elsevier 2017.

The VS_2_ nanofibers facilitated adsorption of large electrolyte amounts as well as provided greater electron movement. It is also noteworthy that the morphology of the fabricated VS_2_ nanoparticles changed from nanofibers at 140 °C to nanosheets at 180 °C, and finally, nanopetals at 200 °C. This was attributed to supersaturation and crystal growth becoming higher as temperatures increased. In order to determine how the morphology of the synthesized nanoparticles affected electrolyte adsorption and the overall counter electrode performance, electrochemical measurements were undertaken. Results from cyclic voltammetry (CV) analysis of the synthesized nanoparticles are illustrated in Figure 9. 

**Figure 10:**
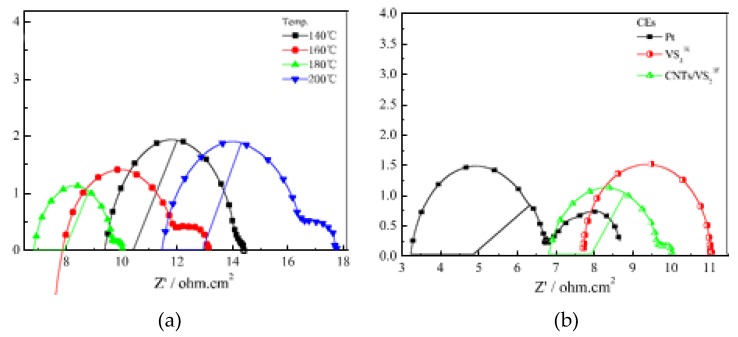
Nyquist plots of (**a**) VS_2_ samples synthesized at different temperatures and (**b**) VS_2_/CNT, VS_2_, and Platinum CEs. Reproduced with permission from Reference [39], Elsevier 2017.

As illustrated in Figure 9a, peak cathodic reduction current density for the VS_2_ nanoparticles decreased in the order 180 °C > 160 °C > 200 °C > 140 °C signaling that maximum catalytic activity was attained at the optimum temperature of 180 °C. Furthermore, this work illustrates how surface area perhaps plays an integral part in influencing catalytic activity. At 140 °C, nanofibers which were touted by Li [49] as the ideal particle morphology for maximum productivity were outperformed by nanosheets which offer maximum surface area for electrolyte adsorption. Figure 9b shows the influence carbon nanotubes have on the VS_2_/CNT composite. As clearly shown, VS_2_/CNTs exhibited the highest reduction current density compared to the platinum and VS_2_ CEs. The synergistic effect produced by the greater catalytic effect of VS_2_ and the excellent electrical conductivity of CNTs as well as their large surface area improved the interfacial charge transfer while also increasing the number of catalytically active sites. Resultantly, greater catalytic activity towards I_3_^–^ reduction was experienced when VS_2_/CNT CE was used compared to the VS_2_ and Pt counter electrodes. Further analysis of the catalytic superiority of the VS_2_/CNT CE compared to the Pt and VS_2_ CEs is illustrated in the charge transfer resistance values from the electrochemical impedance spectroscopy analysis results depicted in Figure 10. 

**Figure 11:**
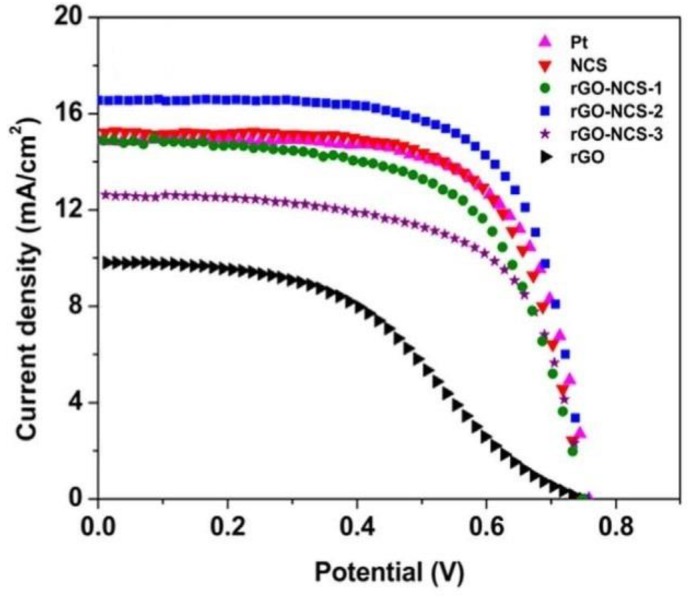
Current-voltage (J–V) characteristics of various DSSCs with different CEs under simulated AM 1 G solar light. Reproduced with permission from Reference [16], Elsevier 2017.

Charge transfer resistance is represented by the 1st semicircle in the high-frequency region of the EIS graph. Ideally, R_ct_ values should be smaller signaling faster electron transfer from the CE to the electrolyte. Variation of charge transfer resistance with an increase in temperature for the VS_2_/CNT counter electrode is illustrated in Figure 10a. At 180 °C, the smallest semicircle can be observed for the VS_2_/CNT CE producing a charge transfer resistance of 2.85 Ω∙cm^2^. From Figure 10b, it is clearly visible that VS_2_/CNT performed better than the Pt and VS_2_ counter electrodes which had 3.65 and 3.37 Ω∙cm^2^, respectively. Therefore, the VS_2_/CNT composite can be deemed to exhibit greater electrical conductivity compared to the other two counter electrodes. The lower charge transfer resistance for VS_2_/CNT was attributed to the excellent electrical conductivity of carbon nanotubes, thereby resulting in less impedance to electron movement. Incorporation of carbon nanotubes with binary VS_2_ to form VS_2_/CNT composites improves the overall effectiveness of the CE, as shown by its higher PCE of 8.02% compared to 6.49% and 6.22% for Pt and VS_2_, respectively. The effect of the carbon support on the functionality of the metal sulphide counter electrode was also recently studied by Sarkar et al. [50] through development of the NiS/rGO counter electrode. Upon incorporation of reduced graphene oxide support on the NiS sample, charge transfer resistance reduced by 50% from 1.5 Ω∙cm^2^ to 3 Ω∙cm^2^, while peak reduction current density increased from 1.4 mA∙cm^2^ to 2.4 mA∙cm^2^. Resultantly, the photo-to-electricity conversion capacity of the DSSC with the NiS/rGO CE was substantially higher at 9.5% compared to 7.7% for the NiS counter electrode, while also being lower than the platinum counter electrode at 9.8% PCE. The higher efficiency parameters for the NiS/rGO electrode were attributed to the excellent distribution of its particles with a 25 nm diameter as well as the excellent conductivity and facilitation of a larger surface area of contact by reduced graphene oxide. Further studies were conducted by Vijaya et al. [51] who developed a MoS_2_/GO-based counter electrode which produced PCE of 8.1% up from 6.8% for the MoS_2_. The platinum counter electrode developed under similar conditions yielded a 6.6% PCE. Zhou et al. [52] also studied the effect of graphene through development of a composite In_2.77_S_4_/GO which yielded a significantly better electron transfer process with charge transfer resistance of 0.57 Ω∙cm^2^ from 96.8 Ω∙cm^2^ for the In_2.77_S_4_ CE. Catalytic activity was also better than the platinum counter electrode with a reduction current density and peak-to-peak potential difference of 3.01 mA∙cm^2^ and 396 mV compared to 2.54 mA∙cm^2^ and 588 mV for platinum. Resultantly, higher photovoltaic parameters were obtained with In_2.77_S_4_/GO yielding a 7.32% PCE as compared to 6.48% and 2.71% for the platinum and In_2.77_S_4_ counter electrodes, respectively. The development of a metal sulphide/carbon composite was further studied by Sun et al. [53] through the fabrication of the CoS/graphene composite. Charge transfer resistance for the developed composite was observed to still be higher than for platinum at 5.9 Ω as compared to 4.1 Ω. Resultantly its PCE was only 5.37% to 5.60 for platinum. Although the efficiency values were still low compared to the silicon-based solar cells, the better performances of the NiS/rGO CE compared to the platinum counter electrode have galvanized researchers towards the development of better counter electrodes. As clearly outlined earlier, the DSSC has to function at levels exceeding that of the silicon-based solar cell as well as cells from other thin-film technologies in the photovoltaic industry. As such, the abovementioned CEs offer insufficient solutions to the efficiency problem in DSSCs, thus, recently, more ternary and quaternary sulphides have been fabricated. Anuratha [16] undertook fabrication of a composite consisting of reduced graphene and the ternary nickel–cobalt sulphide (rGO-NiCoS_4_) counter electrode which exhibited high electrical conductivity. The good electrical and catalytic properties of rGO-NiCoS_4_ were attributed to the synergistic effect between the two materials in the composite as well as the inverse spinel structure of NiCoS_4_, which provides more octahedral catalytic active sites of Co^3+^. The fabricated rGO-NCS counter electrode produced 8.15% PCE as compared to 7.36 and 7.15% for NiCo_2_S_4_ and platinum, respectively. Figure 11 shows the current-voltage (J–V) curves for the DSSCs with various rGO-NiCo_2_S_4_ counter electrodes. 

**Figure 12:**
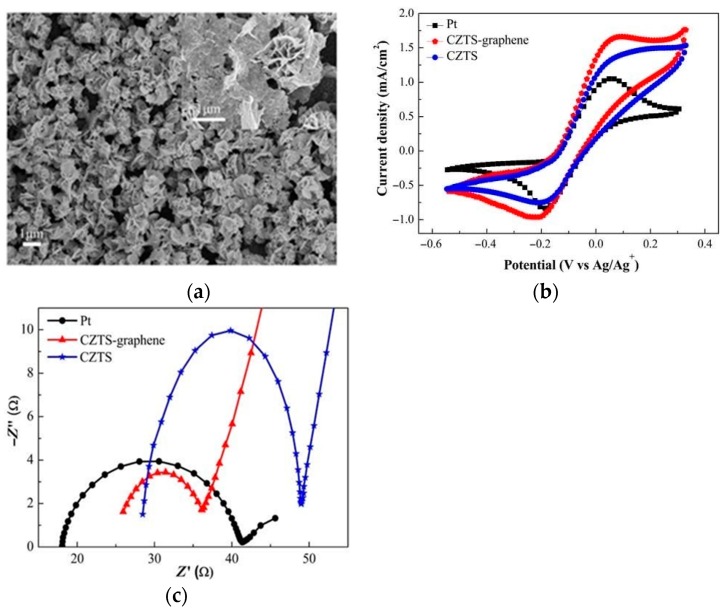
(**a**) SEM, (**b**) CV, and (**c**) EIS micrographs for Pt, Copper-zinc-tin-sulphide (CZTS), and CZTS–graphene. Reproduced with permission from Reference [54], Elsevier 2016.

Open-circuit voltage was almost similar for all the fabricated counter electrodes since it is dependent on the photoanode, which is the same for all the counter electrodes. The higher short-circuit current was the highest for rGO-NCS-2, which was attributed to the superior electrocatalytic capability of this counter electrode. Efficiency parameters obtained for this counter electrode were lower than the best performing carbon-based counter electrode fabricated by Yue [41]. Charge transfer resistance for the best performing rGO-NCS-2 at 0.37 Ω was almost equivalent to the 0.32 Ω for the GCT composite. As clearly shown, the performance of composites composed of ternary metallic sulphides and carbon materials were barely sufficient to replace platinum. The lure of cheaper metallic sulphides with carbon-based materials could be cost effective for mass production. However, currently, research has spilled into fabrication of quaternary metallic–sulphide composites. Li [54] undertook the fabrication of a CuZnSnS_4_–graphene counter electrode for use in DSSCs. The CuZnSnS_4_ (CZTS) being a p-type semiconductor has a large optical absorption coefficient (>10^4^ cm^−1^) as well as excellent electrocatalytic activity. The CZTS–graphene counter electrode was synthesized via a solvothermal method at 230 °C for 24 h. Figure 12a shows the SEM micrographs for the CZTS counter electrode.

**Figure 13:**
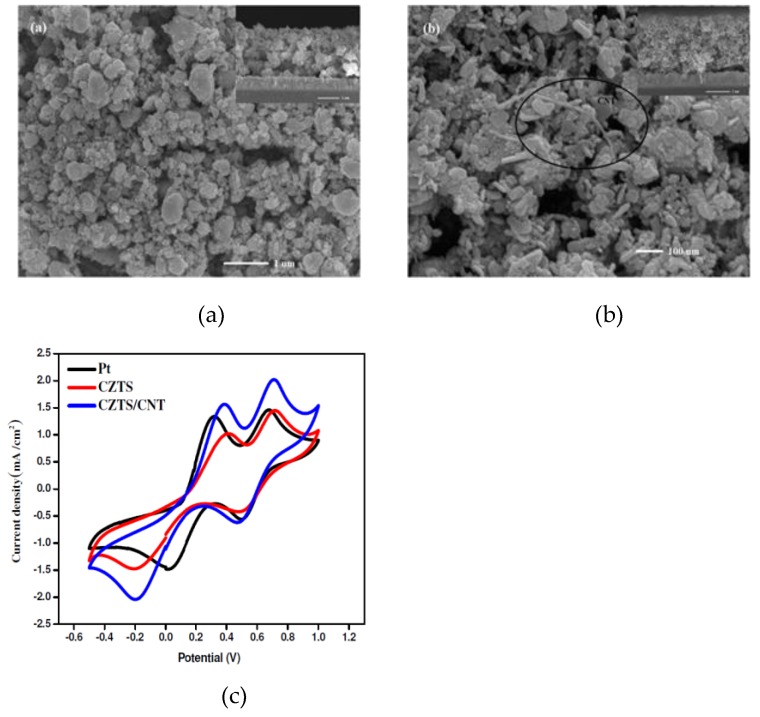
SEM images of (**a**) CZTS nanoparticles, (**b**) CZTS/CNT composite, and (**c**) CV micrograph for the Pt, CZTS, and CZTS/CNT counter electrodes. Reproduced with permission from Reference [55], Elsevier 2016.

It is evident from Figure 12a that CZTS particles formed nanosheets which were vital in widening surface area coverage, thereby improving electrolyte interaction with the CE. Cyclic voltammetry analysis carried out with a three-electrode system are shown in Figure 12b. It was clear that the CZTS–graphene CE, despite its efficient properties, was less catalytically active than the Pt counter electrode. This was derived from the higher reduction current density for the Pt CE as compared to CZTS–graphene. Resultantly, the charge transfer resistance for CZTS–graphene was higher than that of platinum. Nyquist plots in Figure 12c, however, contradict this point, with a larger semicircle for Pt and CZTS compared to CZTS–graphene. The lower charge transfer resistance for CZTS–graphene was attributed to the higher specific surface of the CZTS microspheres and graphene which enlarged the contact area, thereby influencing higher charge transfer. Power conversion efficiencies of the four CEs were 8.12, 7.34, 5.40, and 4.83% for the Pt, CZTS-–graphene, CZTS, and graphene, respectively. In this case, it was evident that, despite facilitating greater electron movement thereby increasing the rate of reduction, PCE hinges more on the operations of the other components of the DSSC than the CE. The CZTS/CNTs synthesized by Nemala [55] with 6.8% PCE outperformed the platinum counter electrode which had 6.3% efficiency. The CZTS–graphene with 7.34% efficiency fared better than CZTS/CNTs in contradiction to Table 2, which describes carbon nanotubes as being superior to graphene. Since the methods of synthesis were different, definitive comparison between the two can only be attained with reference to the platinum (Pt) CEs, which were fabricated simultaneously with each CZTS counter electrode. The CZTS/CNTs synthesized via a solid-state reaction produced microspheres which exhibited aggregation, as shown in Figure 13a. This could potentially have been a drawback to the effectiveness of the CZTS/CNT counter electrode, since aggregation reduces surface accessibility, thereby limiting interaction between the electrolyte and the counter electrode. 

Cyclic voltammetry measurements depicted in Figure 13c show that CZTS/CNT had a greater peak reduction current density to the Pt and CZTS counter electrodes. The unavailability of electrochemical data for CZTS/CNT makes it difficult to analyze and validate which of the two counter electrodes performed better. However, according to the PCE values, CZTS–graphene performed better than the CZTS/CNT counter electrode. Other notable composites from multi-metallic sulphides have found wider use in supercapacitors. Shen [56] developed a CoNi_2_S_4_/CNT/graphene nanocomposite which was touted as inducing remarkable electrochemical properties. The EIS analysis for the CoNi_2_S_4_/CNT/graphene counter electrode showed that in the lower frequency region, CoNi_2_S_4_/CNT/graphene exhibited lower R_ct_ compared to CoNi_2_S_4_–graphene signifying greater catalytic activity when carbon nanotubes were introduced in the composite. The effective catalytic work of CoNi_2_S_4_/CNT/graphene was attributed to the desirable nanosheet structures these particles exhibit. Table 5 shows the performance of varied sulphide composites that have been developed up to date.

The efficiencies of most sulphide/carbon composite counter electrodes are still very low. Charge transfer resistance for most of the analyzed composites is still very high, despite being incorporated with extremely conductive carbon materials such as carbon nanotubes, graphene, and reduced graphene oxide. Furthermore, ternary and quaternary sulphide composites which were believed to enhance catalytic capability through their spinel structures do not exhibit any significant advantage over the binary metallic sulphide composites. The efficiency of the best performing sulphide/carbon composite CZTS/CNT counter electrode is lower than the graphene/CNT (GCT) counter electrode. Charge transfer resistance for these two composites showed that the CZTS/CNT exhibited an extremely large impedance of 4.19 Ω∙cm compared to GCT, which had 0.32 Ω∙cm. The superior catalytic capability of GCT was also indicated by its higher current density of 20.76 mA∙cm^−2^, while the CZTS–CNT counter electrode managed only 16.62 mA∙cm^−2^. The performances of these counter electrodes were lower than the best performing platinum-based counter electrode, which possessed a 13.8% PCE [7]. Although efficiencies and electrochemical properties for all metal sulphide/carbon counter electrodes are still lacking as compared to the platinum electrode, the continuous research and development of existing technologies provides a promising future towards development of a suitable replacement to the platinum counter electrode. 

## 4. Conclusion

The dye-sensitized solar cell offers a plausible alternative to the expensive silicon-based solar cells. However, the low photo-to-current conversion efficiency as well as the high cost of the platinum counter electrode in the DSSC makes it a cost ineffective alternative. Research towards the development of alternatives to the platinum counter electrode has led to the fabrication of varied materials exhibiting varied photo-to-current conversion efficiency. Amongst the feasible alternatives are sulphides, which individually produced modest photovoltaic parameters, as indicated by the PCEs for CoMoS_X_ and CoNiMoS_x_ at 9.64% and 9.12%. Composites of metallic sulphides and carbon materials were observed to also perform well due to the synergy between the two materials, which facilitated greater catalytic activity as well as excellent electrical conductivity. Binary NiS/rGO Ternary rGO-NiCo_2_S_4_ and quaternary CZTS–CNT produced efficiencies of 9.5%, 8.15%, and 9.04%, respectively, which were lower than the 13.8% for platinum. Incorporation of carbon materials into sulphide composites was shown to enhance the electrical conductivity of the composite as indicated by the reduction of charge transfer resistance from 0.85Ω∙cm^2^ for NiCo_2_S_4_ to 0.37Ω∙cm^2^ for rGO-NiCo_2_S_4_. Furthermore, composites of only carbon materials were shown to perform better than all of the analyzed metallic sulphide/carbon composites. The graphene/carbon nanotube composite (GCT) with 10.96% PCE offers a better alternative to all the counter electrodes. As clearly indicated, the efficiency of all the developed counter electrodes were just slightly less than the platinum efficiency, thus little improvements could lead to higher efficiencies. In that regard, more research to develop effective counter electrodes should be conducted.

## 5. Recommendation

As clearly outlined in this review, metallic sulphides and their composites with carbon materials have efficiencies slightly less than the expensive platinum counter electrode. More research is required to produce an effective metal-sulphide counter electrode. The most feasible approach to developing materials which could potentially produce higher catalytic effectiveness and photovoltaic parameters than platinum is through manipulating their morphology such that they offer sufficient area for catalytic activity and facilitate efficient electron transportation as shown by the work conducted by Sarkar [50], Qian [36], and Xu et al. [37]. Further improvements can be achieved by the incorporation of higher performing carbon-based composites such as the graphene/carbon nanotube composite with 10.96% PCE. The higher efficiency of the graphene/carbon nanotube counter electrode offers a feasible approach in which it could partake in composites with metallic sulphides so as to shore-up electrical conductivity and facilitate efficient electron transfer to the oxidized dye, thereby eliminating the potential of electron hole recombinations.

## Figures and Tables

**Table 1:** Photovoltaic parameters of various binary sulphide counter electrodes.

Sample	Synthesis Method	R_ct_/Ω∙cm^−1^	R_s_/Ω∙cm^−1^	J_sc_/mA∙cm^−2^	V_oc_/V	ɳ/%	Ref.
NiCo_2_S_2_	Solvothermal	3.40	11.12	17.17	0.690	7.43	[23]
SnS_2_	Solvothermal	26.3		9	0.68	2.82	[24]
CoS	Hydrothermal	2.09	9.95	16.81	0.747	7.16	[25]
FeS_2_	Hydrothermal	5.99	2.91	12.08	0.74	5.78	[26]
CoS	Electrophoretic deposition	1.05	18.9	16.50	0.757	7.72	[27]
NiCo_2_S_4_	Solvothermal	3.40	11.12	17.17	0.690	7.43	[23]
NiCo_2_S_4_	Hydrothermal	4.00	10.6	17.40	0.743	8.50	[10]
CuInS_2_	Bath deposition	4.42	20.49	12.48	0.780	5.79	[28]

**Table 2:** Properties of various carbon-based materials. Reproduced with permission from Reference [18], Elsevier 2016.

Material	Advantages	Disadvantages
Carbon nanotube	High electrical conductivity, large surface area	Low quantities of defective sites
Graphene	Remarkable carrier mobility, high electrical conductivity	Susceptibility to oxidative environments, low defective sites
Carbon black	Numerous defective sites, high surface-area-to-volume ratio	Poor conductivity
Graphite	Excellent conductivity and corrosion resistance	Low surface area

**Table 3:** Photovoltaic parameters for various carbon-based materials.

Sample	Synthesis Method	R_ct_/Ω∙cm^−1^	R_s_ /Ω∙cm^−1^	J_sc_/mA∙cm^−2^	V_oc_/V	ɳ/%	Ref.
Graphite		2.190	8.170	15.80	0.767	8.478	[40]
CNT-graphene	hydrothermal	0.32	12.88	20.76	0.760	10.56	[41]
MWCNT-lipase	coating	1.39	8.83	14.57	0.693	7.52	[42]
MWCNT/Charcoal	acid functionalization	0.60	5.40	15.90	0.714	8.42	[43]
Graphite		1.54		16.59	0.700	7.88	[44]
Carbon black	doctor blading	0.54		15.19	0.770	8.29	[45]
rGO	doctor blading	5.39	30.72	9.89	0.690	4.04	[46]
Graphene/CNT	doctor blading	1.10	27.80	8.80	0.770	4.00	[47]
Graphene	acid functionalization	750	12.9	8.9	0.700	2.50	[48]

MWCNT—Multi walled carbon nanotubes. CNT—Carbon nanotubes. rGO—Reduced graphene oxide

**Table 4:** Prices of metallic sulphides and carbon-based materials.

Material/g	Sigma–Aldrich/Rands	Cheaptubes/Rands
SWCNT	1010	156–300
MWCNT	129	20
Graphene	1188	2–30
Graphite	50.25	
Carbon black	32.40	
Reduced Graphene oxide	660	190–200
CoS_2_	10.88	
MoS_2_	0.414	
FeS_2_	0.2544	
Platinum	275–614	

SWCNT-single walled carbon nanotubes.

**Table 5:** Photovoltaic parameters for various metallic sulphide/carbon composite CEs.

Sample	Synthesis Method	R_ct_/Ω∙cm^−1^	R_s_ /Ω∙cm^−1^	J_sc_/mA∙cm^−2^	V_oc_/V	ɳ/%	Ref.
MoS/CNF	Solvothermal	2.50	11.70	15.64	0.771	8.40	[57]
MoS/GO	Electrodeposition	1.99	2.50	16.96	0.722	8.01	[49]
SnS/GO	Solvothermal	19.12		13.28	0.690	3.91	[24]
VS_2_/CNT	Hydrothermal	2.85	6.83	15.57	0.755	8.02	[39]
CZTS–graphene	Solvothermal	13.33	25.90	27.15	0.711	8.39	[54]
CZTS–CNT	Solvothermal	4.19	19.22	16.62	0.760	9.04	[55]
rGO-NiCo_2_S_4-1_	Solvothermal	1.11	9.81	14.90	0.750	7.06	[16]
rGO-NiCo_2_S_4-2_	Solvothermal	0.37	9.71	16.40	0.752	8.15	[16]
rGO-NiCo_2_S_4-3_	Solvothermal	1.32	11.1	12.50	0.746	6.01	[16]
rGO-NiCo_2_S_4_	Co-precipitation	2.81	20.56	11.66	0.742	6.01	[58]
CZTS-MWCNT	Solvothermal	4.19	19.22	16.62	0.760	9.04	[59]
